# Mice Lacking GD3 Synthase Display Morphological Abnormalities in the Sciatic Nerve and Neuronal Disturbances during Peripheral Nerve Regeneration

**DOI:** 10.1371/journal.pone.0108919

**Published:** 2014-10-16

**Authors:** Victor Túlio Ribeiro-Resende, Tiago Araújo Gomes, Silmara de Lima, Maiara Nascimento-Lima, Michele Bargas-Rega, Marcelo Felipe Santiago, Ricardo Augusto de Melo Reis, Fernando Garcia de Mello

**Affiliations:** 1 Instituto de Biofísica Carlos Chagas Filho, Universidade Federal do Rio de Janeiro, Laboratório de Neuroquímica, Rio de Janeiro, Rio de Janeiro, Brazil; 2 Instituto de Biofísica Carlos Chagas Filho, Universidade Federal do Rio de Janeiro, Laboratório de Neurobiologia Celular e Molecular, Rio de Janeiro, Rio de Janeiro, Brazil; 3 Núcleo Multidisciplinar de Pesquisa em Biologia - NUMPEX-BIO, Universidade Federal do Rio de Janeiro, Pólo de Xerém, Duque de Caxias, Rio de Janeiro, Brazil; Boston Children's Hospital and Harvard Medical School, United States of America

## Abstract

The ganglioside 9-O-acetyl GD3 is overexpressed in peripheral nerves after lesioning, and its expression is correlated with axonal degeneration and regeneration in adult rodents. However, the biological roles of this ganglioside during the regenerative process are unclear. We used mice lacking GD3 synthase (Siat3a KO), an enzyme that converts GM3 to GD3, which can be further converted to 9-O-acetyl GD3. Morphological analyses of longitudinal and transverse sections of the sciatic nerve revealed significant differences in the transverse area and nerve thickness. The number of axons and the levels of myelin basic protein were significantly reduced in adult KO mice compared to wild-type (WT) mice. The G-ratio was increased in KO mice compared to WT mice based on quantification of thin transverse sections stained with toluidine blue. We found that neurite outgrowth was significantly reduced in the absence of GD3. However, addition of exogenous GD3 led to neurite growth after 3 days, similar to that in WT mice. To evaluate fiber regeneration after nerve lesioning, we compared the regenerated distance from the lesion site and found that this distance was one-fourth the length in KO mice compared to WT mice. KO mice in which GD3 was administered showed markedly improved regeneration compared to the control KO mice. In summary, we suggest that 9-O-acetyl GD3 plays biological roles in neuron-glia interactions, facilitating axonal growth and myelination induced by Schwann cells. Moreover, exogenous GD3 can be converted to 9-O-acetyl GD3 in mice lacking GD3 synthase, improving regeneration.

## Introduction

Gangliosides are glycolipids from a broad family of molecules, and they play extensive biological roles in vertebrate cells, including neurons [1]. Among these gangliosides, 9-O-acetyl GD3 is well known for its role during the development of nerves and dorsal root ganglia (DRG) [2], [3]. The GD3 moiety is the direct precursor of 9-O-acetyl GD3 and is acetylated by the GD3-specific 9-O-acetyltransferase. Once 9-O-acetyl GD3 is incorporated in the lipid portion of the plasma membrane, this molecule is involved in cell division, motility, extension and death during the development and regeneration of the peripheral nervous system (PNS) and central nervous system (CNS) [4]. Expression of 9-O-acetyl GD3 is detected in migrating neurons, growing axons and proliferating Schwann glia during development [5]. A marked reduction in its expression is also found during late stages of PNS development. The addition of GD3 to CHO-K1, 293T cells or human skin fibroblasts, which lack this molecule, leads to its rapid adsorption by the plasma membrane and its conversion to the ganglioside 9-O-acetyl GD3 [6], [7].

Immunoinhibition using antibodies specific to components of the 9-O-acetyl GD3 pathway alters several neuronal processes, including cell migration, both *in vitro* and *in vivo* [8], [9]. However, it is not clear whether animals exhibit a normal phenotype or altered neural function in the absence of 9-O-acetyl GD3 ganglioside. Additionally, during development, 9-O-acetyl GD3 expression is upregulated in the sciatic nerve after lesion via compression, with its expression peaking on day 7 [10]. Moreover, growing axons of cultured DRG neurons display a high expression level of 9-O-acetyl GD3, particularly at the tips of growth cones. Active growth cones collapse when treated with a monoclonal antibody against 9-O-acetyl GD3, via a mechanism that involves microtubule depolymerization [2], [11], [12]. 9-O-acetyl GD3 can be associated with the integrin-β1 subunit in the growth cones of DRG neurons. The interaction between integrin-β1 and laminin-2 in peripheral neurons promotes Ca^2+^ influx, which facilitates neurite outgrowth [14], [15]. However, it not known whether the absence of 9-O-acetyl GD3 modifies integrin-β1 expression or impairs Ca^2+^ influx in growing axons.

The association of 9-O-acetyl GD3 with β-distroglycan in non-myelinated cells or with the Erb-B2 receptor in myelinated cells has been reported to mediate bacterial internalization during infection of Schwann cells by *Mycobacterium leprae* [16]. This association was also detected during severe demyelination of sensory axons [17]. Therefore, we examined whether the lack of 9-O-acetyl GD3 could be related to axon myelination during development.

After addressing the involvement of GD3 and its acetylated conformation in the cellular mechanisms described above, we investigated whether peripheral nerves (including their morphology, degeneration and regeneration) are altered in GD3 synthase-null mice. Here, we report that the absence of this ganglioside interferes with the proper development of mouse sciatic nerves by reducing axonal number and myelin thickness. Our results indicate that GD3 is required for the proper growth and myelination of developing and regenerating axons.

## Materials and Methods

### Animals

Wild-type (129SVEV) mice of either sex were purchased from Jackson Laboratory, and GD3 synthase-null mice (background 129SVEV) generated by Kawai et al. (2001) were a gift from Dr. Steven Walkley (Department of Neuroscience, Albert Einstein College of Medicine). All animals were maintained in the transgenic mouse facility at the Biophysics Institute of Universidade Federal of Rio de Janeiro. All animal handling and experimental protocols were approved by the Animal Care and Use Committee of the Biophysics Institute (CEUA protocol: IBCCF #158).

### Behavioral testing

Both WT (N = 10) and GD3s KO mice (N = 10) were submitted to a sensory test (formalin test) and to a locomotor test (rotarod). In the formalin test, the mice were injected with 20 µl of 1% formalin [37% (w/w) formaldehyde solution, diluted in distilled water] into the subplantar space of the right hind paw. The test was carried out in a transparent plastic chamber of 30×30×30 cm with a mirror placed at the base of the chamber to allow clear observation and an unobstructed view of the mouse. After formalin injection, the mouse starts licking and biting the formalin-injected paw. This behavior is observed in two phases: the first phase, which occurs within 0–1 min (pain threshold), and the second phase, which occurs between 2 and 5 min (pain from swelling). Between these two phases, the animal does not show licking behavior [34]. The total time (seconds) spent in licking and biting the injected paw in both phases was measured. Hot plate test was carried out for the assessment of pain sensitivity. The plate was heated to 52°C and the mice were confined there by a plexiglass cylinder (diameter 15 cm, height 20 cm). Latency (s) to show hind paw response (licking or shaking) was measured. The cut-off time was set at 120 s to avoid tissue damage and prolonged animal suffering. Motor ability and coordination were evaluated using a Rotarod apparatus (Orchid Scientifics, Maharashtra, India) for mice (25 cm length, 4 cm diameter). The ability of young adult mice to keep their balance on a rotating bar at 11, 26 and 37 rpm was tested during 3-min trials, and the fall latency was measured in both GD3s KO and WT mice. Mice underwent two trials separated by 15 minutes.

### Surgical procedures

Sciatic nerve (SN) crush was performed using fine smooth forceps (#5, Fine Tools Instruments) at mid-tight level in male mice under anesthesia using xylazine chloride (8 g/kg Rompum 0.5%, Bayer, São Paulo, SP, Brazil) and ketamine chloride (80 g/kg Vetaset 5%, Fort Dodge Laboratories, Overland Park, KA). No bleeding or muscle lesions were observed after the surgical procedure. After recovering from anesthesia, the animals were returned to the animal facility and housed with free access to water and food. This crush model was employed for Wallerian degeneration and regeneration analysis.

### Ganglioside preparation

We used purified GD3 (5 mg/mL, Matreya, LLC) to analyze the potential adsorption capacity of regenerating nerves and cultured neurons. After adding 5 mg of GD3 to 1 mL of pure DMEM without serum, the suspension was sonicated for 5 minutes, forming micelles with lipid sites exposed on the surface. The final concentration for injection at the lesion site or administration to the culture medium was 20 µg/mL or 5 µg/mL, respectively.

### Tissue preparation

Mice were anesthetized and perfused transcardially with paraformaldehyde (PF) solution (4% PF in 0.1 M phosphate buffer, pH 7.4) at 5, 7, 14, 21 or 42 days after crush lesion. The sciatic nerves were removed, cryoprotected in 30% sucrose overnight and mounted using Tissue-Tek O.C.T. Compound (Sakura Finetechnical, Tokyo, Japan). Frozen longitudinal or transverse sections of SNs were sliced at 16 µm using a cryostat (Leica CM, 1850) and mounted on gelatin pre-coated slides. The slides were then stored at −20°C and protected against humidity.

### Cell and tissue culture

DRG explant cultures for neurite growth assay were obtained from WT (N = 4) or GD3s null (N = 8) P1 mouse embryos. Pups were sacrificed via decapitation in a CO_2_ chamber, and the DRGs were carefully removed immediately afterward. Then, the samples were incubated in DMEM/F-12 supplemented with 50 ng/mL NGF (Life Technologies) for 1 h at 36°C and 5% CO_2_ before plating on coverslips pre-coated with 100 µg/mL of poly-L-lysine (Sigma) and 50 µg/mL of laminin (Invitrogen). DRG explants from both groups of animals were incubated for 5 days and then washed once with 10 mM PBS, fixed using PF 4% and immunostained using antibodies against Tuj-1 (Covance, Cat# MRB-435P-100, RRID: AB_10175616) or integrin-β1 (Abcam, Cat# ab52971, RRID: AB_870695). Of the 8 GD3s null DRG explants cultured, at two days after incubation, 4 explants were co-incubated with micelles containing GD3 ganglioside (Matreya) for three additional days.

DRG explants were also obtained from P1 mouse embryos for calcium imaging. DRGs were dissected and incubated in DMEM/F-12 supplemented with 50 ng/mL NGF (Life Technologies) for 1 h at 37°C in 5% CO_2_. The DRGs were cleaned and incubated at 37°C for 10 min in 0.05% trypsin in Ca^2+^- and Mg^2+^-Free Hanks' solution (CMF). After centrifugation and removal of the trypsin solution, the DRGs were washed with 10 mL of DMEM containing 10% FBS and triturated using a fire-polished Pasteur pipette. Neurons and glia were seeded at low density on poly-L-lysine- (10 µg/ml) and laminin- (20 µg/ml) coated 4-well dishes (Nunc Inc., Rochester, NY, USA). The culture conditions were the same as described above for the DRG explants. The neurons were incubated at 37°C in a humidified 5% CO_2_ incubator for 48 h. At least three independent counts were performed for each experimental paradigm. Neurite growth was assessed via confocal microscopy after immunostaining for Tuj-1. Variations in the free intracellular calcium level ([Ca^2+^]i) were evaluated in DRG neurons or Schwann cells obtained from P1 DRGs following an adaptation of the protocol reported by de Melo Reis et al., 2011 [18]. DRG neurons and Schwann cells were seeded on poly-L-lysine- and laminin-coated coverslips for 48 h. The cells were loaded for 40 min with 5 µM Fura-2/AM (Molecular Probes) in Krebs solution (132 mM NaCl, 4 mM KCl, 1.4 mM MgCl_2_, 2.5 mM CaCl_2_, 6 mM glucose, 10 mM HEPES, pH 7.4) containing 0.1% fatty acid-free bovine serum albumin (BSA) and 0.02% pluronic acid F-127 (Molecular Probes) in an incubator containing 5% CO_2_ and 95% atmospheric air at 37°C. After a 10-min post-loading period at room temperature in Krebs solution, to attain complete hydrolysis of the probe, the 15 mm glass coverslip (Marienfeld, Germany) containing the cells was mounted on an RC-20 chamber in a P-5 platform (Warner Instruments, Hamden, CT) on the stage of an inverted fluorescence microscope (Eclipse Ti-U; Nikon). The cells were continuously perfused with Krebs solution and stimulated according to the protocol. Solutions were added to the cells by a gravity perfusion system, including a set of syringes and a rotary selector valve. The variations in [Ca^2+^]i were evaluated at intervals of 500 milliseconds, by quantifying the ratio of the fluorescence emitted at 510 nm following alternate excitation at 340 and 380 nm, using a Lambda DG4 illumination system (Sutter Instrument, Novato, CA), a 40× objective and a 510 nm band-pass filter (Semrock, Rochester, NY) before fluorescence acquisition with a 16-bit cooled EMCCD camera (Photometrics, Tuscon, AZ). Acquired values were processed using MetaFluor software (Molecular Devices, Sunnyvale, CA). Values for Fura-2 fluorescence ratio were calculated based on a threshold of 20% increase in the [Ca^2+^]i level induced by the stimulus.

### Immunofluorescence

For single or double immunostaining using KI-67 or KI-67/GFAP, frozen sections were equilibrated at room temperature (RT) in a humidified chamber. The slides were then placed in a 4% PF wet chamber for 30 min to promote adhesion of the sections to the slides. Next, the slides were washed twice for 5 min each with 10 mM PBS, pH 7.4, at RT prior to 30 min incubation in 0.01 M citrate buffer, pH 6.0, at 95°C. Then, the slides were washed three times with 10 mM PBS, pH 7.4, containing 0.3% Triton X-100. After these steps, the slides were incubated in 5% normal goat serum (NGS, Life Technologies) in wash solution for 1 h at RT. Incubation with the primary antibodies rabbit monoclonal anti-KI-67 (Abcam, Cat# ab15580, RRID: AB_443209) and mouse monoclonal anti-GFAP (Dako, Cat# Z0334, RRID: AB_10013382) was performed overnight at 4°C, followed by 3 washes with 10 mM PBS containing 0.3% Triton X-100 (5 min each). Then, the sections were incubated with the appropriate secondary antibodies (Alexa Fluor 488-conjugated goat anti-mouse, Life Technologies, Cat# A10680, RRID: AB_11180055, and Cy3 goat anti-rabbit, Jackson ImmunoResearch Labs, Cat# 111-006-045, RRID: AB_10015288) for 2 h at room temperature. After 3 washes, the sections were mounted using Vectashield containing DAPI (Vector Labs) and analyzed using an epifluorescence microscope (Axiovert 200M, Zeiss) or a confocal microscope (LSM 510, Zeiss). Longitudinal and transverse sections of the sciatic nerve, as well as DRG explant cultures, were also fixed using 4% PF, gently washed 3 times with 10 mM PBS containing 0.1% Triton X-100 and incubated in 10% (slices) or 5% (explants) NGS for 1 h, followed by incubation with monoclonal rabbit anti-NF-200 (Sigma, Cat# N4142, RRID: AB_477272) for 2 h. The samples were washed 3 times with PBS and incubated with anti-rabbit Alexa 488 (Life Technologies, Cat# A21206, RRDI: AB_10049650). Axonal density and neurite outgrowth were assessed via confocal microscopy (LSM 510, Zeiss). For F4-80 (activated mouse macrophages) immunofluorescence (Serotec, Cat# MCA497, RRID: AB_2098196), the procedures were the same, except that Triton X-100 was excluded from the PBS and anti-rat Alexa 555 (Life Technologies, Cat# A21434 RRID: AB_10562898) was used as the secondary antibody. Immunofluorescence of transverse sections of the sciatic nerve for myelin basic protein (Santa Cruz Biotechnology, Cat# sc-25665, RRID: AB_648796) Santa Cruz Biotechnology, Inc.) and the endothelial isoform of nitric oxide synthase (eNOS), (Sigma, Cat# SAB4502016, RRID: AB_10744644) was performed in a manner similar to that described for the longitudinal sections. Longitudinal sections of the sciatic nerve and the DRG explant cultures were also double immunolabeled for integrin-β1 (Abcam, Cat# ab52971, RRID: AB_870695) and either GD3 (Abcam, Cat# ab11779, RRID: AB_298562) or 9-O-acetyl GD3 (CD-60b) (Santa Cruz Biotechnology, Cat# sc-32269, RRID: AB_627135).

### Semi-thin sections

The animals were anaesthetized using ketamine (100 mg/kg) and xylazine (15 mg/kg) and euthanized via transcardial perfusion using a fixative solution (4% PF and 2% glutaraldehyde in 0.1 M phosphate buffer (PB), pH 7.4). After perfusion, the sciatic nerve was collected. The middle segments of the nerves were immersed for 2 h in a solution of 2.5% glutaraldehyde in 0.1 M PB (pH 7.4), washed with 0.1 M cacodylate buffer (pH 7.4) and post-fixed for 90 minutes in 1% osmium tetroxide containing 0.8% potassium ferrocyanide and 5 mM calcium chloride in 0.1 M cacodylate buffer (pH 7.4). The segments were then washed in 0.1 M cacodylate buffer (pH 7.4) and stained with 1% uranyl acetate overnight in the dark. The next day, the nerves were dehydrated using increasing concentrations of acetone (from 30% to 100%), infiltrated with Embed-812 resin (Electron Microscopy Sciences) and polymerized at 60°C for 48 h. Semi-thin (500 nm) transverse sections were generated using an RMC MT-6000 ultramicrotome. The semi-thin sections were stained with 1% Toluidine Blue and examined using a light microscope (Zeiss Axioskop 2 Plus).

### Quantitative analyses and statistics

Cells stained for NF-200, DAPI, MBP, F4-80, p-histone H3, GAP-43, integrin-β1 and KI-67/GFAP were counted in optical sections via confocal microscopy (LSM 510 Meta, Zeiss, Germany). *In vitro* neurite extension from the DRG neurons was assessed in the images using Axiovision 4.3 software (Carl Zeiss, Germany), which was also used to count the number of proliferating Schwann cells. To quantify the total number of myelinated fibers in each nerve, photographs of the semi-thin cross sections were captured via light microscopy. Five fields from each semi-thin cross section were analyzed at a magnification of 100×. For each sample, we calculated and compared the following parameters in both groups: nerve fiber area, axon area, myelin area and G-ratio. The myelin area was measured by subtracting the axon area from the fiber area. The G-ratio was calculated by dividing the axon diameter by the fiber diameter, and the results were stratified in ranges of 0.0–0.399, 0.4–0.499, 0.5–0.599, 0.6–0.699, 0.7–0.799 and 0.8–0.899. The mean of G-ratio for each nerve (5 per group) was plotted and analyzed to compare WT and GD3s KO mice.

Statistical analyses were performed using one-way analysis of variance (ANOVA) followed by a Newman-Keuls post-test to compare all pairs of experimental conditions for three or more conditions. When two experimental conditions were analyzed, we performed the *Mann-Whitney* test. All data are expressed as the means ± standard error of the mean (SEM). The symbols in the histograms are as follows: *, *p*<0.01; **, *p*<0.001 and ***, *p*<0.0001.

## Results

### Adult mice lacking GD3s display reductions in axonal density and myelination

**Figure 1:**
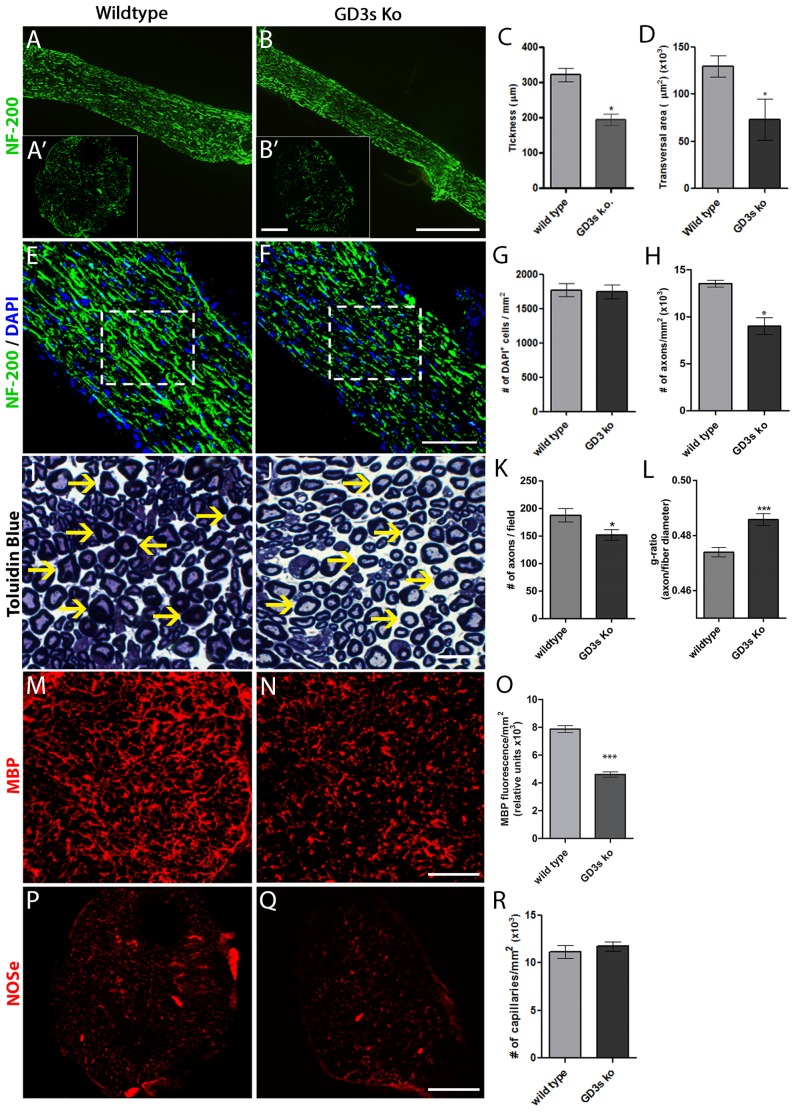
Morphological analysis of the sciatic nerve in adult mice lacking GD3s. A, B, E and F: Longitudinal sections of unlesioned sciatic nerves from adult mice immunostained for NF-200 at low (A and B) and high magnifications (E and F) from WT (A and E) or GD3s KO (B and F) mice. A′ and B′: Cross-sections of the same samples immunostained for NF-200. The nuclei were counterstained with DAPI. I, J, M, N, P and Q: Cross-sections of sciatic nerves immunolabeled for toluidine blue (I, J semi-thin sections), myelin basic protein (M and N) or the endothelial isoform of nitric oxide synthase (P and Q). Yellow arrows in I and J indicate myelin thickness. C, D, G, H, K, L, O and R: Histograms of quantitative analysis related to the counts or densitometry represented in the images. Statistics: *Mann-Whitney* * *p<0.01*; *** p<0.0001. Bars: A, B = 500 µm; A′, B′, M, N = 50 µm; E, F, P, Q = 100 µm; and I, J = 25 µm.

Longitudinal and transverse sections of the sciatic nerve immunolabeled for NF-200 are shown for both WT and GD3s KO mice (Fig. 1A, B, A′ and B′). Quantitative analyses comparing nerve thickness and the transverse area revealed that the nerve thickness and the transverse area of the GD3s KO mice were one-third smaller compared to WT mice (Fig. 1C and D). No difference in cell density was detected between the two groups [Fig. 1E, F (dashed squares) and G]. However, the number of axon puncta was 32% lower in the mice lacking GD3s ganglioside (Fig. 1H). The G-ratio of GD3s KO mice revealed a reduced myelin thickness compared to WT mice [Fig. 1I, J (yellow arrows) and L). Additionally, a 30% reduction in axon fibers was found in mice lacking GD3s (Fig. 1K). Immunostaining for myelin basic protein (MBP) in adult nerve cross-sections revealed reduced levels of this protein in GD3s KO mice based on confocal microscopy (Fig. 1M, N and O). This result confirms the reduction in the G-ratio described above. There was no difference in the number of capillaries between WT and GD3s KO mice (Fig. 1P, Q and R).

### Mice lacking GD3s display sensory and motor dysfunction

**Figure 2:**
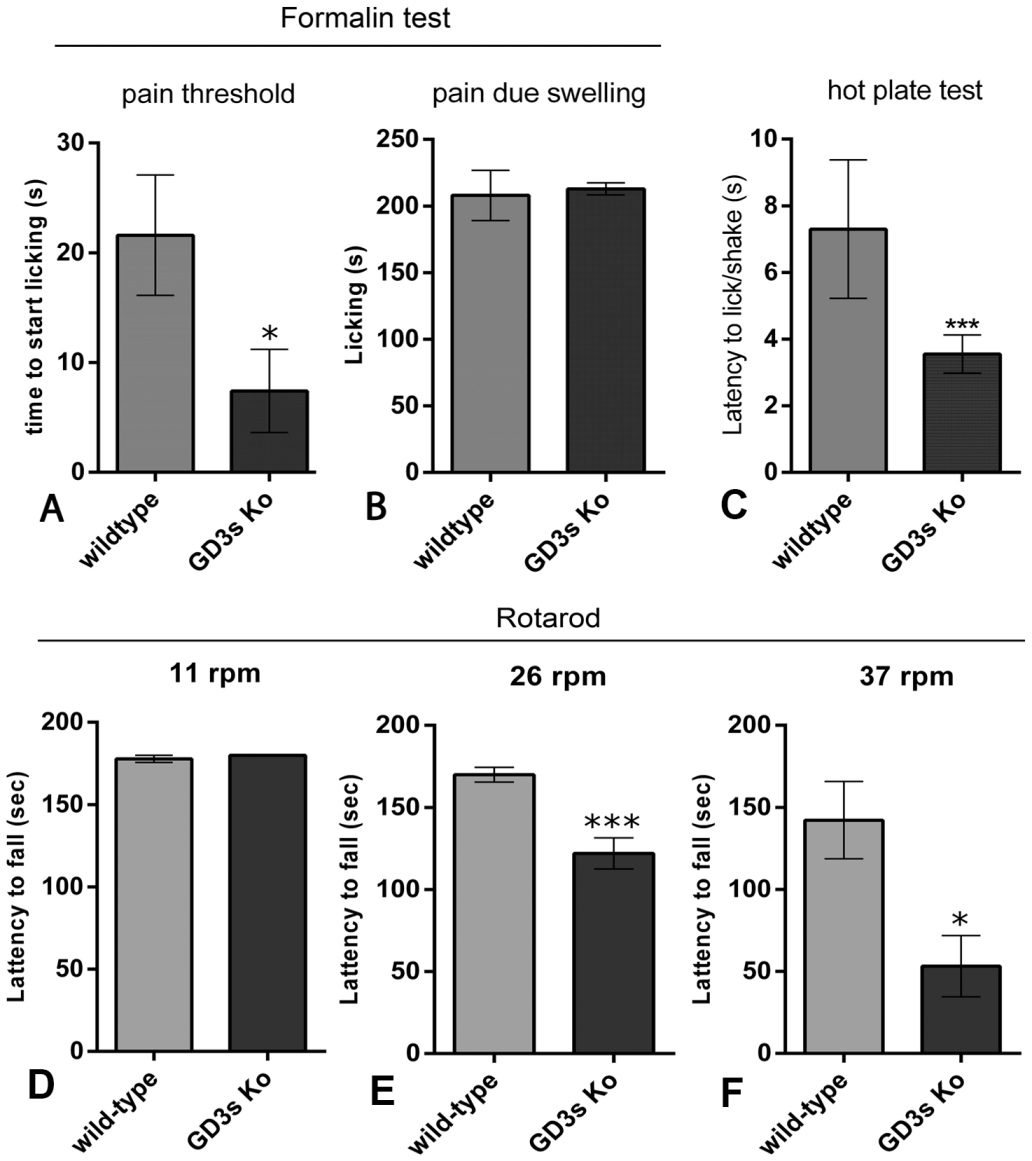
Sensory and motor behaviors of mice lacking GD3s. A–B: Histograms indicating the time to initiate licking (A) and licking duration (B) after injection of 5 µL of 2% formalin into the right forepaw (n = 10 for each group). C: Histogram indicating the latency of mice to initiate licking on a hot plate apparatus at 52°C (N = 10 for each group). D–F: Histograms indicating the motor behavior performed on the rotarod apparatus, on which mice were challenged daily at 11 rpm (C), 26 rpm (D) and 37 rpm (E). Statistics: *Mann-Whitney* * p<0.01; *** p<0.0001.

We evaluated sensory and motor activities associated with axons present in the sciatic nerve. The pain threshold was assessed by measuring the initiation of licking behavior (from 0 to 180 seconds) after injection of 20 µL of 0.5% formalin at the dorsal side of the foot paw. Mice lacking GD3s exhibited a 1.5-fold reduction in the pain threshold (Fig. 2A), but there was no difference in the pain level caused by swelling between WT and KO mice (Fig. 2B). This result suggests that the absence of GD3 ganglioside and its acetylated form affects the threshold to painful stimuli, but once triggered, there is no difference in the duration of the response. To evaluate a possible hyperalgesic behavior of mice lacking GD3s we proceed the hot plate test at 52°C measuring the latency to lick or shake paws. In fact, these animals exhibited 50% reduction in the latency compared to the wildtype mice (Fig. 2C, N = 10 per group, *p<0,0001 Mann-Whitney*). This observation corroborates with reduced pain threshold described above and strongly suggests the hyperalgesic behavior of mice lacking GD3s.

GD3s KO animals exhibit normal social behavior and motivation for food and water. We performed the rotarod locomotor test at various velocities (11, 26 and 37 rpm). At 11 rpm, both WT and GD3s KO mice maintained walking for the same period (Fig. 2D, N = 5 per group, *p = 0.75 Mann-Whitney*). When the speed was increased to 26 rpm, mice lacking GD3s reduced their pace on the wheel compared to the WT mice (Fig. 2E, N = 5, per group, *p<0.0001 Mann-Whitney*). The difference between the groups increased further at 37 rpm (Fig. 2F, N = 5 per group, *p<0.01 Mann-Whitney*). We conclude that at medium (26 rpm) or high (37 rpm) speeds, motor deficits are notable in KO mice compared to WT mice. Therefore, mice lacking GD3s display both sensory and motor alterations when stimulated, but no differences were detected under normal living conditions.

### Wallerian degeneration (WD) is not altered in mice lacking GD3s after nerve crush lesioning

**Figure 3:**
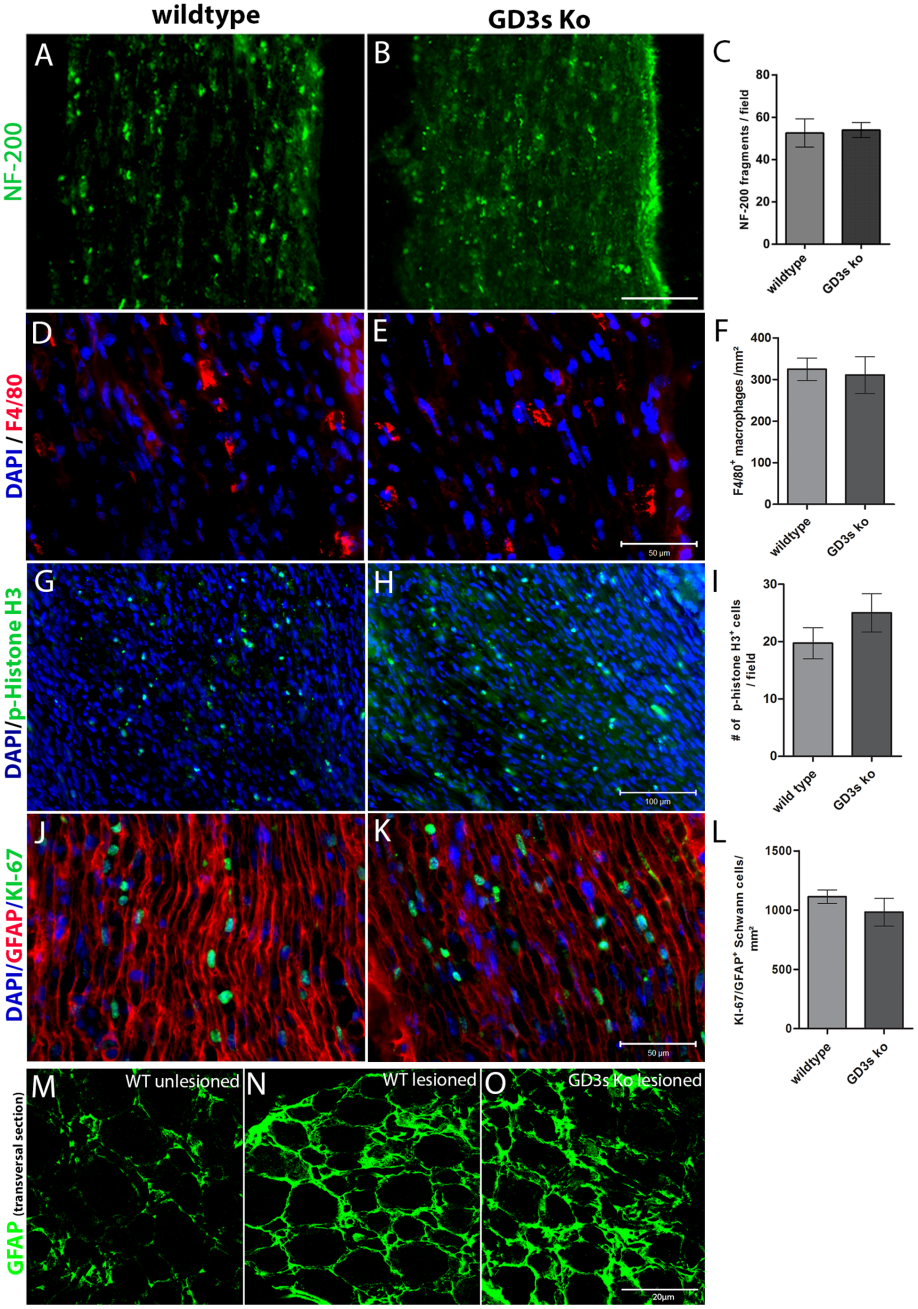
Wallerian degeneration in mice lacking GD3s: tissue infiltration by activated macrophages and Schwann cell proliferation. A–D: Longitudinal sections of lesioned sciatic nerves from adult WT and GD3s KO mice at 5 days after crush lesioning immunolabeled for NF-200 (A, B) or F4-80 (C, D) and imaged at the distal nerve stump. The nuclei were counterstained with DAPI. E: Histogram indicating the number of active macrophages (cells positive for F4-80) at the distal nerve stump. G–H; J–K: Longitudinal sections of lesioned sciatic nerves from WT and GD3s KO mice imaged at the distal stump 7 days after crush lesioning and immunolabeled for p-histone H3 (G–H) or double immunolabeled for Ki-67 and GFAP (J–K). The nuclei were counterstained with DAPI. I and L: Histograms indicating the number of cells positive for p-histone H3 (I) or KI-67/GFAP (L) at the distal stump in each group of mice. M–O: Optical slices obtained by confocal microscopy from transversal sections of wildtype uninjured (M) and injured (N) or GD3s Ko injured sciatic nerves immunolabeled for GFAP at distal stump, 5 days after crush lesion. Bars: A–B, G–H = 100 µm; and D–E, J–K = 50 µm; M–O = 20 µm. Statistics: *Mann-Whitney*, *ns, p>0.05*.

It is known that 9-O-acetyl GD3 expression is upregulated in Schwann cells during early steps of peripheral nerve regeneration (Ribeiro-Resende et al., 2007). Therefore, we analyzed the temporal profile of WD at the sciatic nerve distal stump of mice lacking GD3 synthase. Axonal fragments immunolabeled for NF-200 (green dots) were detected at the nerve distal stump five days after crush lesioning (Fig. 3A and B). Quantitative analysis of these dots demonstrated no significant difference between the GD3s KO and WT mice (Fig. 3C). Next, we compared active macrophage invasion in the same region (Fig. 3D and E). Indeed, macrophages were present in nerve slices from both groups. No difference was detected in the number of activated macrophages between the two groups (Fig. 3F).

Schwann cell proliferation is another crucial step for axon regeneration along injured peripheral nerves. We compared the total cell proliferation at the distal stumps of the sciatic nerves in both groups via immunofluorescence for p-histone H3 (Fig. 3G and H). There was no difference in the percentage of cell proliferation between the GD3s KO and WT mice (Fig. 3I). Sections immunostained for KI-67 and GFAP to detect Schwann cell proliferation (Fig. 3J and K) also revealed no differences in the crushed nerves from the two mouse strains (Fig. 3L). Compared to the unlesioned nerve tissue by conventional fluorescence, GFAP expression is increased in both WT and GD3s KO mice five days after lesion (Fig. 3M and N). In this set of experiments, nerves are undergoing a degenerative process where cell density and extracellular matrix have been modified by Wallerian degeneration. For this reason we were not able to detect differences of thickness in degenerating/regenerating nerve tissue as we did for the unlesioned nerves. Taken together, these results suggest that the absence of GD3/9-O-acetyl GD3 did not compromise the degenerative process of the peripheral nerves.

### Sciatic nerve regeneration in GD3s KO is partially restored by exogenous GD3

**Figure 4:**
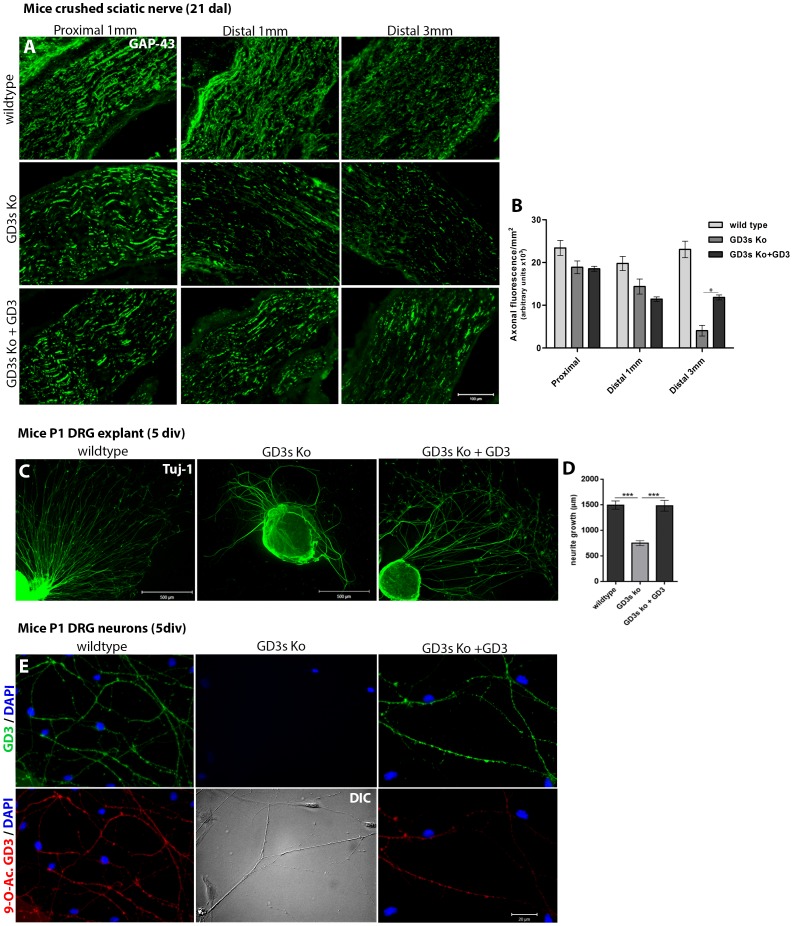
Committed nerve regeneration in adult mice lacking GD3s is restored by administration of exogenous GD3 *in vivo* and *in vitro*. A–I: Longitudinal sections of sciatic nerves proximally or 1 mm or 3 mm distally immunolabeled for GAP-43 at 21 days after crush lesioning. J: Histogram indicating the axonal density in the regenerating nerves from WT, GD3s KO and GD3-treated GD3s KO mice. K–M: Images of P1 mouse DRG explants seeded on PDL/laminin coverslips. The DRG samples from WT, GD3s KO and GD3s KO exogenously treated with GD3 ganglioside were incubated for 5 days *in vitro*. GD3 was added on day 2 of the incubation. Low-magnification images of DRGs immunolabeled for Tuj-1. N: Histogram quantifying neurite growth. O–T: High-magnification images of neurites immunolabeled for R24 (GD3, O–Q) or CD-60b (9-O-Acetyl GD3, R–T). The nuclei were counterstained with DAPI (white) Bars: A–I = 100 µm; K–M = 500 µm; and O–T = 20 µm. Statistics: ANOVA *** p<0.001; * *p<0.01*.

For these experiments, we injected 10 µL of GD3 ganglioside in micelles (5 µg/µL) into the lesion site immediately after crush lesioning. Then, we analyzed longitudinal sections of the nerves immunolabeled for GAP-43 three weeks after lesioning. As shown in Fig. 4A, WT mice had a significantly higher axonal density compared to the GD3s KO mice or the KO mice treated with GD3 micelles (Fig. 4A and B). At the distal stump (1 mm from the lesion site), the WT mice displayed an axonal density 25% greater than that of the GD3s KO mice. Administration of exogenous GD3 had no effect on the axonal density (Fig. 4A and B). However, when the axonal density 3 mm from the lesion site was measured, administration of GD3 induced a 2-fold increase in the axonal density compared to the GD3s KO mice (Fig. 4A and B). We compared neurite outgrowth of cultured DRG neurons from WT mice, GD3s KO mice and GD3s KO mice treated with GD3 micelles in the presence of NGF (50 ng/mL). Neurite extension was reduced by half in DRGs from the GD3s KO mice (Fig. 4C, D). Additionally, treatment of the culture medium with exogenous GD3 (5 µg/µL) induced neurite extension similar to that of WT DRGs after 72 h (Fig. 4 C and D). However, the number of neurites did not differ. Both GD3 and 9-O-acetyl GD3 were detected after double immunofluorescence staining for R24 (GD3) and CD-60b (9-O-acetyl GD3) in WT neurites (Fig. 4E, left panel). As expected, there was no fluorescence signal for either marker in GD3s KO neurons, despite the presence of neurites shown by DIC microscopy (Fig. 4E, middle panel). However, GD3- and 9-O-acetyl GD3-positive neurites were detected three days after incubation with exogenous GD3 micelles (Fig. 4E, right panel). These results suggest that mice lacking GD3s exhibit a reduced potential for neuronal growth and regeneration. Exogenous GD3 was partially converted to 9-O-acetyl GD3. This treatment alleviates this neuronal dysfunction, allowing neurites to grow in a manner similar to that of neurons from WT mice.

### Integrin-β1 subunit but not calcium influx is altered in DRG neurons lacking GD3s

**Figure 5:**
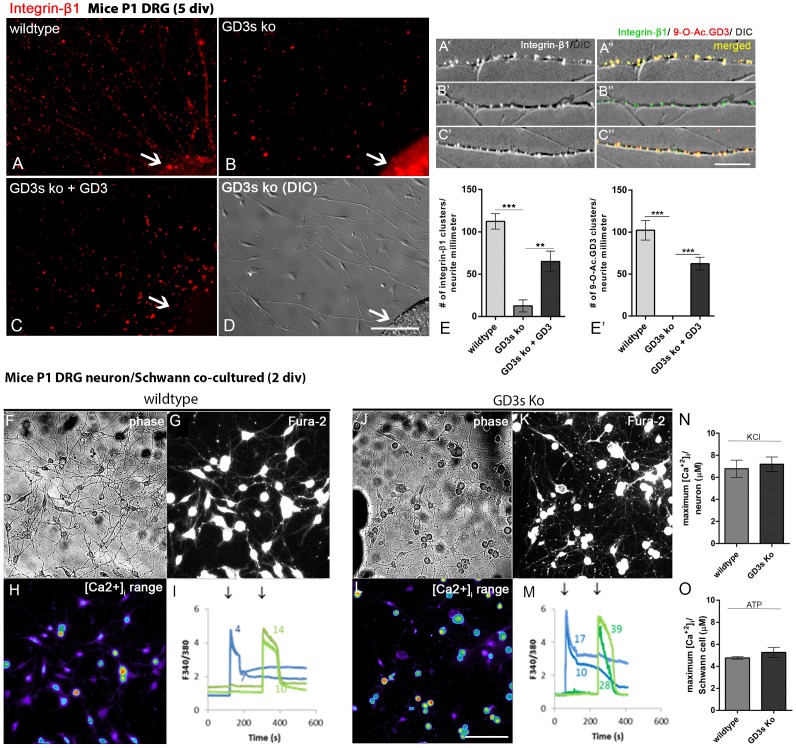
Integrin-β1 expression but not calcium influx is modified in neurons lacking GD3s. A–D: Images of integrin-β1 obtained by apotome microscopy of mice neonate (P1) DRGs from WT (A), GD3s KO (B) and GD3s KO with exogenous GD3 (C). Samples were cultured for 5 days. A′–C′: High-magnification optical sections of neurites (DIC) double-labeled for integrin-β1 and CD60-b (9-O-ac. GD3). Yellow dots indicate colocalization of the two markers. D: DIC image of the field shown in C, illustrating DRG (lower right) and neurites extended. E, E′: Histogram of quantitative analysis of the number of integrin-β1 (E) or 9-O-Ac. GD3 (E′) clusters along extended neurites. F and J: P1 postnatal DRGs were dissected, cleaned, dissociated and cultured for 48 h in the presence of 50 ng/ml NGF. Cell cultures from both WT (F) and GD3s KO (J) mice show typical neurons with extensive neurites and flat Schwann cells. The same fields are shown under fluorescence (G, K). Further, H and L show the same microscope field under fura-2 fluorescence, in SCCI experiments. Typical responses are shown for 4 cells in WT (I) or in GD3s KO mice (M) when stimulated with 50 mM KCl (blue, first arrow) or 100 µM ATP (green, second arrow). As shown in WT (F, G, H), cells #4 and #7 are neurons (with large cell bodies), whereas cells 14 and 10 are flat, typical of Schwann glia. The same is observed for GD3s KO cells (J, K, L), where cells #17 and #10 are neurons, and cells #28 and #39 are glia. N, O: Histograms indicating maximum calcium influx when stimulated by KCl (O, neurons) or ATP (P, Schwann cells). Bars: A–D = 500 µm; A′–C′ = 100 µm F–H, J–L = 20 µm. Statistics: ns, p = 0.760, *** p<0.0001 Mann-Whitney; * p<0.001 ANOVA.

The absence of GD3s preferentially affects neurons, which show a reduction in the capacity to extend neurites *in vitro* as well as the growth and regeneration of axons in mouse sciatic nerves. For this reason, we first analyzed the expression of integrin-β1 in DRG neurons from P1 mice. There was a dramatic reduction in the integrin-β1 concentration in neurites from samples lacking GD3 synthase compared to those from WT mice (Fig. 5A, A′, B, B′ and E). Curiously, the fluorescence intensity of integrin-β1 was remarkably higher in GD3s KO DRGs compared to WT DRGs (Fig. 5A and B, arrows). DIC microscopy showed that DRGs from the KO mice extended neurites (Figure 5D), excluding the possibility that reduced expression of the integrin-β1 subunit was due to the absence of growing neurites. Exogenous GD3 administered to the DRGs from KO mice was adsorbed and partially restored the levels of integrin-β1 expression in the neurites (Fig. 5C, C′ and E, E′), and it reduced the fluorescence intensity of the DRGs (Fig. 5C, arrow). Moreover, 9-O-acetyl GD3 derived from exogenous GD3 was observed to colocalize with integrin-β1, as detected in neurites from WT DRGs (Fig. 5A″ and C″, yellow dots). These results suggest that integrin expression is not reduced but that the transport of integrin-β1 from the soma to the neurites likely fails, leading to an accumulation of this protein in the neuronal cell bodies. The lack of normal integrin-β1 levels in neurites might explain the weakness of neuronal projections found in the absence of GD3/9-O-acetyl GD3 ganglioside (Fig. 5A′ and B′). Therefore, neuronal adhesion to extracellular matrix elements, such as laminin, might facilitate axonal growth.

To evaluate the functional responses of cultured DRG cells (neurons and Schwann cells) from WT and GD3s KO mice, intracellular Ca^2+^ variations were measured following application of 50 mM KCl or 100 µM ATP using a method adapted from a previous study of retinal cells [18]. The assumption was that KCl would depolarize neurons because it is known that L-type calcium channels are expressed in postnatal primary sensory neurons [19]. However, purinergic receptors are broadly expressed in all major classes of glia, including Schwann cells. Therefore, ATP should selectively activate these cells [20]. Both WT (Fig. 5F) and GD3s KO (Fig. 5J) mouse cell cultures display typical neurons containing neurites and flat Schwann cells. Equivalent fluorescence microscopic fields are shown for WT (Fig. 5G) and GD3s KO cultures (Fig. 5K). Additionally, Figures 5H and L show the Fura-2 fluorescence of selected cells in the same microscopic fields; typical responses are shown for 4 cells from WT (Fig. 5I) or GD3s KO mice (Fig. 5M) stimulated with 50 mM KCl (blue, first arrow) or 100 µM ATP (green, second arrow). As shown in the WT culture (Fig. 5F–I), cells numbered 4 and 7 are neurons (with large cell bodies), whereas cells 14 and 10 display the typical flat morphology of Schwann glia. The same results were found for GD3s KO cells (Fig. 5J–M), in which cells 17 and 10 are neurons, and cells 28 and 39 are glia. Neurons from both WT and KO mice showed increased calcium influx when stimulated with KCl (Fig. 5N). In addition, Schwann cells from both experimental groups displayed the same level of calcium influx when stimulated with 100 µM ATP, including the maximum values (Fig. 5O). In conclusion, no differences were observed in the responses of neurons or Schwann cells when we analyzed the calcium influx in co-cultures from both WT and GD3s KO mice (Fig. 5 J and N). Taken together, these results reinforce the evidence that mice lacking GD3 synthase display reduced neuronal function due to altered molecular parameters of cell adhesion. Moreover, physiological responses involving Ca^2+^ influx are preserved in GD3s KO mice.

## Discussion

In the present study, we analyzed the morphology, Wallerian degeneration and regeneration of the PNS in mice lacking GD3s, an enzyme that converts GM3 to the ganglioside GD3. In all of these processes involved in the development or regeneration of peripheral axons, we found a preferential disturbance of DRG neurons followed by Schwann cells, as both the number of nerve fibers and the amount of myelination were reduced in adult KO mice (Fig. 1). These mice did not display any motor or sensory disturbance under normal housing conditions, but when they were challenged, they showed both motor and sensory deficits. This result could be associated with previously observed changes in neuronal morphology.

Gangliosides comprise a broad family of glycolipids that attach to cell membranes, including the plasma membrane. These molecules can bind to several types of receptors and channels, facilitating the stabilization and the functional conformation of these proteins [21], [22]. Special attention has been devoted to the ganglioside 9-O-acetyl GD3, the production of which requires GD3s. This ganglioside is generated via the simple acetylation of GD3 by 9-O-acetylase. Immunoinhibition of 9-O-acetyl GD3 in DRG mouse embryos (E16) reduces neuritogenesis by collapsing growth cones [2]. It is well established that this ganglioside binds to integrin-β1 subunits in neurons [15], but the mechanisms involved in the weakness of peripheral axons remain unclear. Here, we found a correlation between the absence of 9-O-acetyl GD3 and a strong reduction in the concentration of the integrin-β1 subunit in neurites (Fig. 5). Administration of exogenous GD3 to DRG neurons partially restored integrin-β1 expression, which correlated well with the recovery of neuritogenesis.

Integrin receptors are cell membrane dimers formed of α and β subunits, and they are expressed as various isoforms. Laminins from the extracellular matrix (ECM) are well known to mediate the binding of molecules to integrins, leading to massive calcium influx into the cytoplasm. Increased levels of cytoplasmic calcium trigger actin dynamics and the motility of growth cones, facilitating axonal extension along chemical pathways during development and regeneration [23], [24]. We examined calcium influx under physiological conditions in mouse DRG neurons lacking GD3s and found no major alterations in the responses of calcium channels when the cultures were incubated in KCl or ATP. This result suggests that 9-O-acetyl GD3 regulates integrin assembly by promoting β1 subunit expression/trafficking along the growing neurites. Therefore, DRGs induced to depolarize might lose the ability to regulate Ca^2+^ entry, resulting in impairments in neurite outgrowth and attachment to the ECM.

Another finding regarding nerve morphology was that the myelin thickness was reduced in adult mice lacking GD3s (Fig. 1). It is well understood that during development and regeneration, peripheral nerve axons and glia control Schwann cell survival and fate, modulating the myelinated or non-myelinated state of the axons [25], [26]. The levels of neuregulin-1 produced by neurons increase with axonal thickness. This factor binds to Erb-B2 receptors on Schwann cells and induces myelination [27]. We found no differences in Erb-B2 expression in Schwann cells or in neuregulin-1 expression in DRG neurons from genetically modified versus WT mice (data not shown). The axonal thickness was not altered despite the reduced number of axons in the nerves lacking GD3s. One possible explanation for the reduction in the myelin thickness may be the reduced number of axons during late steps of nerve development, which would reduce the average neuregulin-1 levels. To better understand the mechanisms underlying this change, further studies are required.

It is well established that the ganglioside 9-O-acetyl GD3 is expressed prenatally by neurons and glia along the course of neural development in rodents [8], [16], [28]. Its expression is strongly reduced on E20, which correlates with the end of axonal growth and the establishment of functional connections between neurons, including the DRG, the lumbar spinal cord and peripheral nerves. Curiously, when axons are lesioned in adult rats, the expression of this molecule is upregulated for a period that correlates to WD and the initiation of axonal regeneration. We found that the peak of ganglioside expression is on day 7 after nerve crush lesioning, and its expression then gradually reduces to basal levels by day 10 after lesioning [10]. We examined selected parameters of WD, including axonal breakdown, macrophage invasion and cell proliferation, specifically including Schwann cell proliferation, at the distal stump. All of these processes acted in synchrony for Schwann cells to form the bands of Büngner, allowing axons to regenerate [29]. Potential changes in the behavior of these cells could represent a possible explanation for the upregulation of 9-O-acetyl GD3 during early steps of nerve regeneration. However, we did not find any difference in WD between GD3s KO and WT regenerating nerves. This result indicates the possibility of preferential neural impairment in mice lacking GD3s. Since, we demonstrated reduced axonal density, demyelination, motor and sensorial dysfunction and slow regeneration we suggest that different types of axonal fibers are committed in these animals.

As we analyzed the regeneration of the adult sciatic nerve, it became clear that the absence of GD3s from axons decreases the axonal growth rate after crush lesioning (Fig. 4). However, administration of exogenous GD3 to the lesion site partially alleviated this effect. DRG neurons in culture demonstrated the same behavior when GD3 was added to the culture. In fact, neurites adsorb GD3 in the membrane, and then GD3 is acetylated to 9-O-acetyl GD3, the molecule that is clearly involved in neuronal migration and axonal growth. These findings support the concept that 9-O-acetyl GD3 is a ganglioside involved not only in neural development [10], [30] but also in regeneration of the PNS. In fact, we cannot exclude the possibility that other gangliosides might participate in nerve regeneration. GD3 is the first ganglioside of the b-series, followed by GD2, GD1b, GT1b and GQ1b gangliosides. GD2 shows no function in neuritogenesis when administered to the GD3s KO DRGs in culture (data not shown). Moreover, GD2 is not acetylated as frequently as GD3, and its acetylation is nearly absent in neurons [31], [32]. In addition, o-series and a-series gangliosides are normally expressed in mice lacking GD3s. This enzyme is downstream of the synthesis of o-series and a-series precursors, which follow different pathways from the b-series gangliosides [33]. Because GM3 is a substrate of GD3s, we expected an accumulation of GM3 in mice lacking GD3s. However, this was not detected, which suggests a negative feedback mechanism involving upstream enzymes [13].

9-O-acetyl GD3 and the broad family of gangliosides are small molecules compared to membrane proteins such as G-protein-coupled receptors, tyrosine kinase receptors or ion channel-coupled receptors. However, the capacity of these glycolipids to stabilize and facilitate the functionality of these receptors has been progressively characterized over time. Particularly because gangliosides are negatively charged due to the presence of sialic acid in their structures, these molecules can act as co-receptors by associating with major proteins or glycoproteins in the cell membrane. Our results help to clarify the biological roles of gangliosides and the mechanisms by which they are involved in neuronal signaling during the early steps of nerve regeneration.
