# Correction: Mice Lacking GD3 Synthase Display Morphological Abnormalities in the Sciatic Nerve and Neuronal Disturbances during Peripheral Nerve Regeneration

**DOI:** 10.1371/journal.pone.0118242

**Published:** 2015-03-04

**Authors:** The PLOS ONE Staff

There are a number of errors in the legends for Figs. 3, 4, and 5. The complete, correct legends for Figs. 3, 4, and 5 are:

**Figure 3. Wallerian degeneration in mice lacking GD3s: tissue infiltration by activated macrophages and Schwann cell proliferation**. A-B; D-E: Longitudinal sections of lesioned sciatic nerves from adult WT and GD3s KO mice at 5 days after crush lesioning immunolabeled for NF-200 (A, B) or F4–80 (D, E) and imaged at the distal nerve stump. The nuclei were counterstained with DAPI. C and F: Histograms indicating the number of NF-200 fragments (C) and active macrophages (cells positive for F4–80) at the distal nerve stump (F). G-H; J-K: Longitudinal sections of lesioned sciatic nerves from WT and GD3s KO mice imaged at the distal stump 7 days after crush lesioning and immunolabeled for p-histone H3 (G-H) or double immunolabeled for Ki-67 and GFAP (J-K). The nuclei were counterstained with DAPI. I and L: Histograms indicating the number of cells positive for phistone H3 (I) or KI-67/GFAP (L) at the distal stump in each group of mice. M-O: Optical slices obtained by confocal microscopy from transversal sections of wildtype uninjured (M), wildtype injured (N) or GD3s Ko injured (O) sciatic nerves immunolabeled for GFAP at distal stump, 5 days after crush lesion. Bars: A-B, G-H = 100 mm; and D-E, J-K = 50 mm; M-O = 20 mm. Statistics: *Mann-Whitney*, *ns*, *p>0*.*05*. doi: 10.1371/journal.pone.0108919.g003

**Figure 4. Committed nerve regeneration in adult mice lacking GD3s is restored by administration of exogenous GD3 in vivo and in vitro**. A: Longitudinal sections of sciatic nerves proximally or 1 mm or 3 mm distally immunolabeled for GAP-43 at 21 days after crush lesioning. B: Histogram indicating the axonal density in the regenerating nerves from WT, GD3s KO and GD3-treated GD3s KO mice. C: Images of P1 mouse DRG explants seeded on PDL/laminin coverslips. The DRG samples from WT, GD3s KO and GD3s KO exogenously treated with GD3 ganglioside were incubated for 5 days in vitro. GD3 was added on day 2 of the incubation. Low-magnification images of DRGs immunolabeled for Tuj-1. D: Histogram quantifying neurite growth. E: High-magnification images of neurites immunolabeled for R24 (GD3, O-Q) or CD-60b (9-O-Acetyl GD3, R-T). The nuclei were counterstained with DAPI (white) Bars: A = 100 mm; C = 500 mm; and E = 20 mm. Statistics: ANOVA ***p<0.001; *p<0.*01*. doi: 10.1371/journal.pone.0108919.g004

**Figure 5. Integrin-β1 expression but not calcium influx is modified in neurons lacking GD3s**. A-C: Images of integrin-β1 obtained by apotome microscopy of mice neonate (P1) DRGs from WT (A), GD3s KO (B) and GD3s KO with exogenous GD3 (C). Samples were cultured for 5 days. A′–C′: High-magnification optical sections of neurites (DIC) labeled for integrin-β1. A″-C″: High-magnification optical sections of neurites (DIC) double labeled for integrin-β1 and CD60-b (9-O-ac. GD3). Yellow dots indicate colocalization of the two markers. D: DIC image of the field shown in C, illustrating DRG (lower right) and neurites extended. E, E′: Histogram of quantitative analysis of the number of integrin- β1 (E) or 9-O-Ac. GD3 (E′) clusters along extended neurites. F and J: P1 postnatal DRGs were dissected, cleaned, dissociated and cultured for 48 h in the presence of 50 ng/ml NGF. Cell cultures from both WT (F) and GD3s KO (J) mice show typical neurons with extensive neurites and flat Schwann cells. The same fields are shown under fluorescence (G, K). Further, H and L show the same microscope field under fura-2 fluorescence, in SCCI experiments. Typical responses are shown for 4 cells in WT (I) or in GD3s KO mice (M) when stimulated with 50 mM KCl (blue, first arrow) or 100 mM ATP (green, second arrow). As shown in WT (F, G, H), cells #4 and #7 are neurons (with large cell bodies), whereas cells 14 and 10 are flat, typical of Schwann glia. The same is observed for GD3s KO cells (J, K, L), where cells #17 and #10 are neurons, and cells #28 and #39 are glia. N, O: Histograms indicating maximum calcium influx when stimulated by KCl (N, neurons) or ATP (O, Schwann cells). Bars: A-D = 500 mm; A′-C′, A′-C″ = 100 mm F-H, J-L = 20 mm. Statistics: *ns*, *p = 0*.760 Mann-Whitney; ***p<0.*0001*, *p<0.*001 ANOVA*. doi: 10.1371/journal.pone.0108919.g005
